# SARS-CoV-2 T Cell Responses Elicited by COVID-19 Vaccines or Infection Are Expected to Remain Robust against Omicron

**DOI:** 10.3390/v14010079

**Published:** 2022-01-02

**Authors:** Syed Faraz Ahmed, Ahmed Abdul Quadeer, Matthew R. McKay

**Affiliations:** 1 Department of Electronic and Computer Engineering, The Hong Kong University of Science and Technology, Hong Kong SAR, China; sfahmed@connect.ust.hk; 2 Department of Chemical and Biological Engineering, The Hong Kong University of Science and Technology, Hong Kong SAR, China; 3 Department of Electrical and Electronic Engineering, University of Melbourne, Parkville, VIC 3010, Australia; 4 Department of Microbiology and Immunology, University of Melbourne, at the Peter Doherty Institute for Infection and Immunity, Parkville, VIC 3000, Australia

**Keywords:** SARS-CoV-2, COVID-19, Omicron, variants, T cell epitopes, mutations, insertions, deletions, peptide-HLA binding

## Abstract

Omicron, the most recent SARS-CoV-2 variant of concern (VOC), harbours multiple mutations in the spike protein that were not observed in previous VOCs. Initial studies suggest Omicron to substantially reduce the neutralizing capability of antibodies induced from vaccines and previous infection. However, its effect on T cell responses remains to be determined. Here, we assess the effect of Omicron mutations on known T cell epitopes and report data suggesting T cell responses to remain broadly robust against this new variant.

## 1. Main Text

Omicron (B.1.1.529), detected in Botswana, South Africa, and Hong Kong in November 2021, has emerged as a new SARS-CoV-2 variant of concern (VOC). Based on SARS-CoV-2 genome data available on GISAID (https://www.gisaid.org (accessed on 25 December 2021)), Omicron has been identified in at least 85 countries, raising concerns about its potentially high transmissibility. The most worrying aspect of Omicron is the abundance of mutations in the spike (S) protein, with some shared with previous VOCs and some new. Preliminary studies have reported a drastic reduction in the neutralization efficacy of infection- and vaccine-elicited antibodies and sera against Omicron [1,2,3,4,5], indicating a strong capability of Omicron to evade humoral immune responses. However, the extent to which Omicron is capable to evade cellular immune responses, the other arm of the adaptive immune system mediated by T cells, is not yet clear.

T cell responses are a key armament against viral infections which, in addition to assisting B cell activation for generating antibodies, help in providing protection from disease by eliminating virus-infected cells. SARS-CoV-2 T cell responses induced by either natural infection or vaccines have been linked to rapid viral clearance and reduced disease severity [6,7,8], even when the neutralizing antibody response is reduced [9] or absent [10]. Thus, if SARS-CoV-2 T cell responses hold up, they are likely to assist in limiting disease severity in infections caused by Omicron that seemingly escapes neutralizing antibodies [1,2,5]. This antibody escape is reported to be facilitated by Omicron-defining mutations present in the N-terminal domain of S, as well as in all four classes of neutralizing antibody epitopes located in the receptor binding domain [3,4]. If mutations in Omicron result in T cell escape, it could also limit the protection provided by T cells.

**Figure 1:**
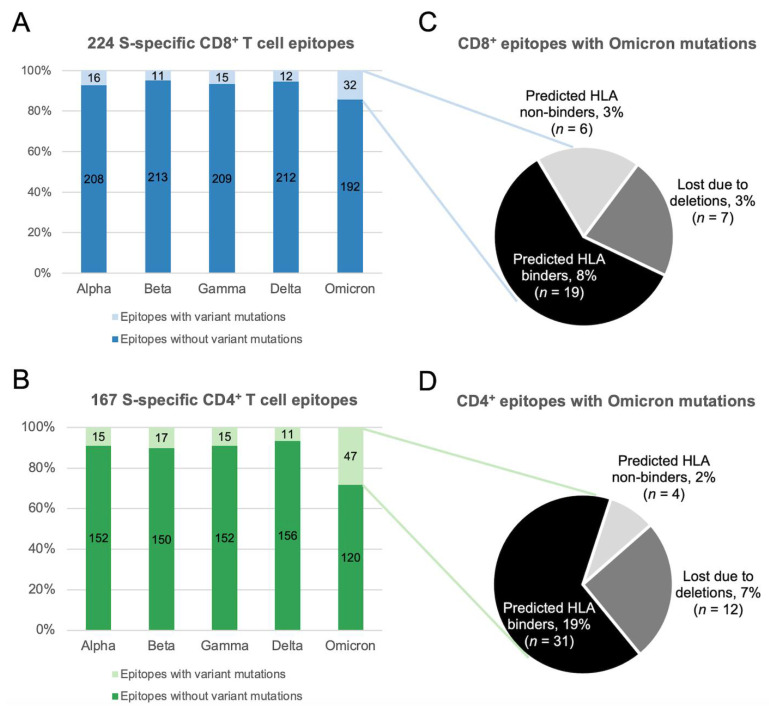
Percentage of S-specific SARS-CoV-2 T cell epitopes with and without mutations present in the five VOCs: (**A**) CD8^+^ T cell epitopes. (**B)** CD4^+^ T cell epitopes. Only epitopes of canonical lengths (CD8^+^: 8–12 residues and CD4^+^:15 residues) were included in the analysis. VOC-defining mutations (that also include deletions) were obtained from https://covariants.org (accessed on 9 December 2021). Predicted effect of Omicron mutations on peptide-HLA binding of SARS-CoV-2 (**C**) CD8^+^ and (**D**) CD4^+^ T cell epitopes. NetMHCpan-4.1 and NetMHCpanII-4.0 were employed for predicting peptide-HLA binding using the default parameters [13].

Here we provide a preliminary investigation into the robustness of T cell responses against Omicron by leveraging information of T cell epitopes known to be targeted in COVID-19 infected and/or vaccinated individuals. We first focused on epitopes derived from the S protein. This is of primary interest in the context of T cell responses elicited by COVID-19 vaccines, with many such vaccines employing S-specific antigens. T cell responses against S have also been shown to be immunodominant upon natural infection [11]. We considered all S-specific 224 CD8^+^ and 167 CD4^+^ SARS-CoV-2 T cell epitopes available at IEDB [12] (accessed on 9 December 2021) and screened them for Omicron-defining mutations (obtained from https://covariants.org (accessed on 9 December 2021)). This revealed that 14% of CD8^+^ and 28% of CD4^+^ T cell epitopes comprise at least one position harbouring an Omicron mutation (Figure 1A,B), indicating that a large majority of both CD8^+^ and CD4^+^ T cell epitopes (86% and 72%, respectively) remain unaffected by Omicron.

The number of epitopes encompassing Omicron mutations (32 CD8^+^ and 47 CD4^+^ epitopes) are nonetheless notably higher than for other VOCs, especially in the case of CD4^+^ T cell epitopes (Figure 1A,B). To further assess the capacity of Omicron mutations to evade responses against these epitopes, we employed widely-used computational tools [13] to predict the impact of Omicron mutations on binding of these S-specific epitopes to their cognate human leukocyte antigen (HLA) alleles. Such HLA-epitope binding is a necessary requirement for T cell recognition. Inspection of these mutations revealed that the multiple deletions in the S protein of Omicron lead to loss of seven CD8^+^ and 12 CD4^+^ epitopes, while only six CD8^+^ and four CD4^+^ epitopes (listed in Tables S1 and S2, respectively) are predicted to become non-binders (Figure 1C,D). In the case of CD8^+^ epitopes, half (3/6) of the epitopes predicted to abrogate HLA binding are exclusive to Omicron, while the remaining were also observed in previous VOCs (Table S1). Since the majority of the CD8^+^ and CD4^+^ epitopes harbouring Omicron mutations are predicted to retain HLA binding (Figure 1C,D), this lowers the possibility of T cell escape. A similar trend regarding the preservation of peptide-HLA binding is also observed for other VOCs (Table S3).

Taken collectively, the presented data would suggest that SARS-CoV-2 T cell immunity acquired by S-focused COVID-19 vaccines or previous infection would remain broadly robust against Omicron, as was the case for other VOCs [14,15].

As an interesting by-product, our analysis revealed that a new peptide EPEDLPQGF, gained for the first time due to the unique three amino acid insertion ‘EPE’ following position 214 in the S protein of Omicron, is predicted to bind strongly with two common HLA alleles (HLA-B*35:01 and B*53:01) and weakly with four others (HLA-A*26:01, B*07:02, B*44:02, and B*51:01). This peptide may constitute a unique Omicron-specific T cell epitope, if confirmed to be targeted.

**Figure 2:**
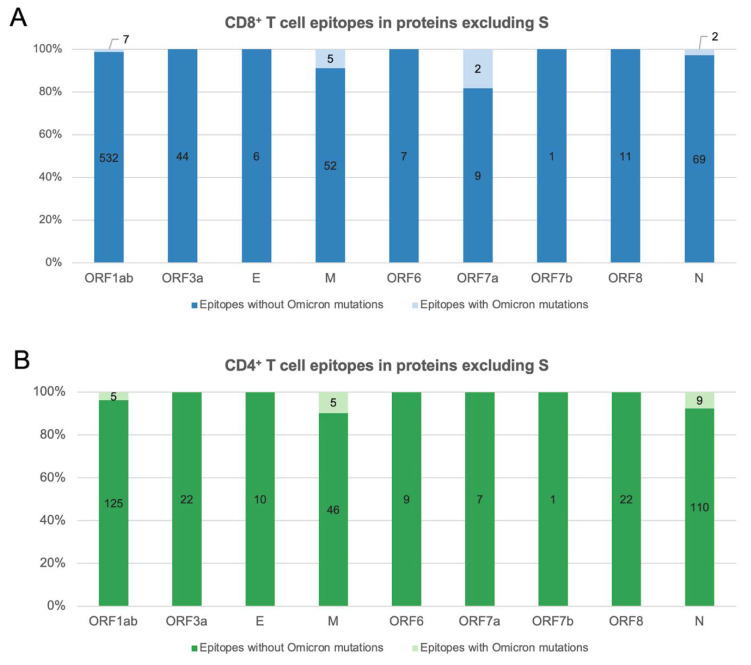
Percentage of SARS-CoV-2 T cell epitopes derived from proteins besides S with and without Omicron-defining mutations: (**A**) CD8^+^ T cell epitopes. (**B**) CD4^+^ T cell epitopes. Only epitopes of canonical lengths (CD8^+^: 8–12 residues and CD4^+^:15 residues) were included in the analysis. Omicron-defining mutations (that also include deletions) were obtained from https://covariants.org (accessed on 9 December 2021).

We next considered SARS-CoV-2 T cell epitopes derived from other proteins, besides S. This is of interest in the context of T cell responses induced by prior natural infection or inactivated COVID-19 vaccines, which have been found to target epitopes derived from multiple SARS-CoV-2 proteins [16,17]. Investigating all 745 CD8^+^ and 373 CD4^+^ SARS-CoV-2 T cell epitopes derived from proteins besides S (available at IEDB [12]; accessed on 9 December 2021) revealed that an overwhelming majority of them (98% and 95%, respectively) remained unaffected by mutations observed in Omicron (Figure 2). Among those affected, deletions accounted for the putative loss of five epitopes, with four (1 CD8^+^ and 3 CD4^+^) being from the immunodominant nucleocapsid (N) protein.

It is the case that some T cell epitopes are preferentially targeted in the population. Escape from responses against the most commonly targeted epitopes should be carefully scrutinized, since it could potentially affect a large fraction of the population. To investigate this, we assessed whether Omicron mutations affect immunoprevalent CD8^+^ T cell epitopes that have been reported to be commonly targeted by convalescent individuals from different geographical regions [18]. As was the case for other VOCs, none of the Omicron mutations were present in any of the 20 immunoprevalent epitopes. Coupling these results with the fact that multiple T cell epitopes are targeted within an individual [11], suggests that the acquired T cell immunity across proteins would be expected to remain largely intact against Omicron.

There are multiple limitations of this study. First, the study is based on a set of experimentally-determined SARS-CoV-2 T cell epitopes that have been reported so far. While this set includes a large number of epitopes, it may not be exhaustive. Second, assessing the robustness of T cell responses against a SARS-CoV-2 variant using the fraction of epitopes encompassing mutations present in the variant is a first order analysis. While it can assist to provide preliminary estimates, further targeted experiments are required to confirm the robustness of T cell responses against Omicron, and also to test the capacity of specific epitope mutations to confer T cell escape. Some initial experimental studies along these lines have already started to appear while this paper was under review, which have arrived at consistent conclusions [19,20,21].

Overall, given that most of the experimental T cell epitopes known to be targeted in vaccinated and/or previously infected individuals (collectively, accounting for ~60% of the global population as of 25 December 2021 [22]) are unaffected by Omicron mutations, our preliminary analysis suggests that the effectiveness of pre-existing T cell immunity will remain intact. T cell responses alone, however, do not block infection and therefore do not prevent transmission. Thus, while the number of infections may rise considerably as a consequence of Omicron’s ability to evade antibodies [23,24], robust T cell immunity provides hope that, similar to other VOCs [25], the level of protection against severe disease would remain high. Emerging data is suggestive of a reduced risk of hospitalisation and death from Omicron [26,27]; however, the degree to which pre-existing T cell immunity is contributing to this still needs to be clearly established.

## Data Availability

Not applicable.

## Supplementary Materials

The following supporting information can be downloaded at: https://www.mdpi.com/article/10.3390/v14010079/s1, Table S1: List of CD8^+^ T cell epitopes with Omicron mutations that are predicted to become non-binders. NetMHCpan-4.1 was employed for predicting peptide-HLA binding using the default parameters; Table S2: List of CD4+ T cell epitopes with Omicron mutations that are predicted to become non-binders. NetMHCpanII-4.0 was employed for predicting peptide-HLA binding using the default parameters; Table S3: Summary of SARS-CoV-2 T cell epitopes affected by mutations in other VOCs. NetMHCpan-4.1 and NetMHCpanII-4.0 were employed for predicting peptide-HLA binding using the default parameters.
