# Supplementary material for: SARS-CoV-2 T Cell Responses Elicited by COVID-19 Vaccines or Infection Are Expected to Remain Robust against Omicron
Source: Viruses. 2022 Jan 2;14(1):79. doi: 10.3390/v14010079 (PMC8781795; doi:10.3390/v14010079)
Supplement: Supplementary file 1 [file viruses-14-00079-s001.zip › viruses-1529279-supplementary.pdf]

SUPPLEMENTARY TABLES

Table S1. CD8+ T cell epitopes harboring Omicron mutations that are predicted to become non-binders. NetMHCpan-4.1 was employed for predicting peptide-HLA binding using the default parameters.

No.	Epitope	HLA	Mutation(s)	VOCs in addition to Omicron
1	FCNDPFLGVYY	A*01:01	Y145D	-
2	YYHKNNKSW	A*24:02	Y145D	-
3	YGFQPTNGV	B*51:01	G496S<br>Q498R<br>N501Y	-
4	QIAPGQTGK	A*68:01	K417N	Beta
5	SPRRARSV	B*07:02	P681H	Alpha
6	SPRRARSVA	B*07:02	P681H	Alpha

Table S2. CD4+ T cell epitopes harboring Omicron mutations that are predicted to become non-binders. NetMHCpanII-4.0 was employed for predicting peptide-HLA binding using the default parameters.

No.	Epitope	HLA	Mutation(s)	VOCs in addition to Omicron
1	CVADYSVLYNSASFS	DQA1*01:01/<br>DQB1*05:03	S371L<br>S373P<br>S375F	-
2	TQLNRALTGIAVEQD	DQB1*04:02	N764K	-
3	NLLLQYGSFCTQLNR	DQA1*01:01/<br>DQB1*05:03	N764K	-
4	CAQKFNGLTVLPPLL	DQB1*06:02	N856K	-

Table S3. Summary of SARS-CoV-2 T cell epitopes affected by mutations in other VOCs. NetMHCpan-4.1 and NetMHCpanII-4.0 were employed for predicting peptide-HLA binding using the default parameters.

No.	VOC	Type	Lost due to deletion(s)	Predicted HLA binder(s)	Predicted HLA non-binder(s)
1	Alpha	$CD8^+$	6	8	2
		$CD4^+$	6	9	0
2	Beta	$CD8^+$	2	8	1
		$CD4^+$	4	13	0
3	Gamma	$CD8^+$	0	13	2
		$CD4^+$	0	15	0
4	Delta	$CD8^+$	2	8	2
		$CD4^+$	1	10	0
